# Ethyl 10α-hy­droxy-4,9-dimethyl-14-oxo-3,8,15-trioxa­tetra­cyclo­[10.3.0.0^2,4^.0^7,9^]penta­decane-13-spiro-5′-pyrazole-3′-carboxyl­ate

**DOI:** 10.1107/S2414314620009451

**Published:** 2020-07-21

**Authors:** Fatima Outahar, Mohamed Moumou, El Mostapha Rakib, Abdellah Hannioui, Mohamed Saadi, Lahcen El Ammari

**Affiliations:** a Laboratory of Organic and Analytical Chemistry, Faculty of Sciences and Technics, Sultan Moulay Slimane University, BP 523, Béni-Mellal, Morocco; b Laboratoire de Chimie Physique et Chimie Biorganique, Faculté des Sciences et Techniques, Université Hassan II, Casablanca, BP 146 Mohammedia, Morocco; c Laboratoire de Chimie Appliquée des Matériaux, Centre des Sciences des Matériaux, Faculty of Sciences, Mohammed V University in Rabat, Avenue Ibn Batouta, BP 1014, Rabat, Morocco; Goethe-Universität Frankfurt, Germany

**Keywords:** crystal structure, hydrogen bonding, furan, pyrazole

## Abstract

The ten-membered ring in the title mol­ecule, C_25_H_29_ClN_2_O_7_, adopts an approximate chair–chair conformation, whereas the five-membered furan and pyrazole rings display envelope conformations. The mean plane of the furan ring is almost perpendicular to that of the pyrazole ring, as indicated by the dihedral angle between them of 86.45 (9)°. The pyrazole ring is slightly inclined to the plane of the attached phenyl ring, subtending a dihedral angle of 16.88 (8)°. The conformation of the mol­ecule is stabilized by six intra­molecular hydrogen bonds and crystal cohesion is ensured by five C—H⋯O hydrogen bonds, in addition to C—H⋯π inter­actions.

## Abstract 2

The ten-membered ring in the title mol­ecule adopts an approximate chair–chair conformation, whereas the five-membered furan and pyrazole rings display envelope conformations. The conformation of the mol­ecule is stabilized by six intra­molecular hydrogen bonds and crystal cohesion is ensured by five C—H⋯O hydrogen bonds, in addition to C–H⋯π inter­action, connecting mol­ecules.

## Graphical abstract

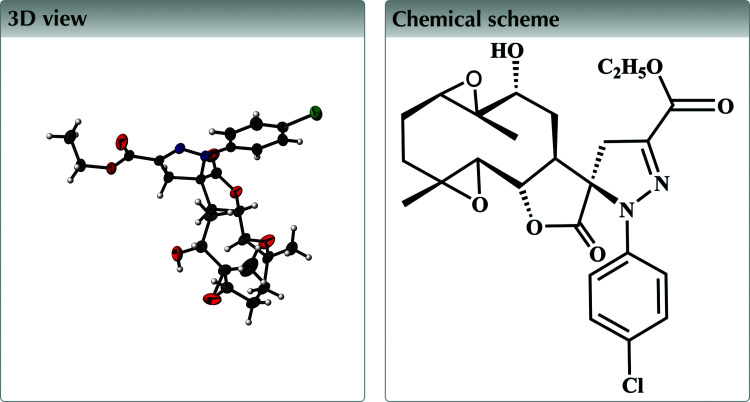

## Structure description

Anvillea radiata is an endemic plant that grows in northern Africa, particularly in the two Maghreb countries Morocco and Algeria. It belongs to the Asteraceae family and is widely used in Moroccan and Algerian traditional medicine for the treatment of dysentery and gastrointestinal disorders (Bellakhdar, 1997 ▸). It also exhibits hypoglycemic activity (Qureshi *et al.*, 1990 ▸), and has been reported to possess anti­tumoral activity (Abdel Sattar *et al.*,1996 ▸). We have previously shown that the aerial parts of anvillea radiata could be used as a renewable source of 9α-hy­droxy­parthenolide (El Hassany *et al.*, 2004 ▸). In order to prepare products with high added value that can be used in the pharmacology and cosmetics industries, we have developed a synthesis of a new spiro-pyrazole by 1,3-dipolar cyclo­addition. Treating 9α-hy­droxy-1β,10α-ep­oxy­parthenolide with 1.2 equivalents amount of *N*-*para*-chloro­phenyl­hydrazono α-bromo­glyoxylate at room temperature gives the title compound ethyl 10α-hy­droxy-4,9-dimethyl-14-oxo-3,8,15-trioxa­tetra­cyclo­[10.3.0.0^2,4^.0^7,9^]penta­decane-13-spiro-5′-pyrazole-3′-carb­oxyl­ate. The structure of this new product was confirmed by single-crystal X-ray diffraction.

**Figure 1:**
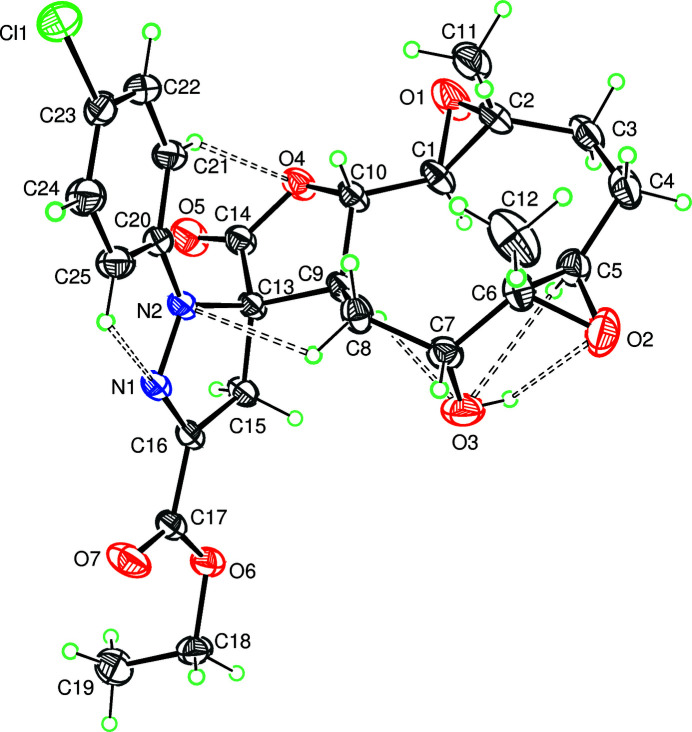
The title mol­ecule with the atom-labelling scheme showing the intra­molecular hydrogen bonds (dashed bonds). Displacement ellipsoids are drawn at the 50% probability level.

The mol­ecule is built up from two fused five- and ten-membered rings, with two additional ep­oxy ring systems and a 4,5-di­hydro-3-phenyl­pyrazole group as a substituent (Fig. 1 ▸). The ten-membered ring adopts an approximate chair–chair conformation, while the pyrazole and the furan rings adopt envelope conformations, with the C13 and C9 atoms as the, respective flaps. The dihedral angle between the mean plan of the pyrazole ring and that of the furan ring is of 86.45 (9)°. The phenyl ring is inclined to the plane of the attached furan ring by a dihedral angle of 16.88 (8)°. The conformation of the mol­ecule is stabilized by six intra­molecular hydrogen bonds (Fig. 1 ▸ and Table 1 ▸).

**Figure 2:**
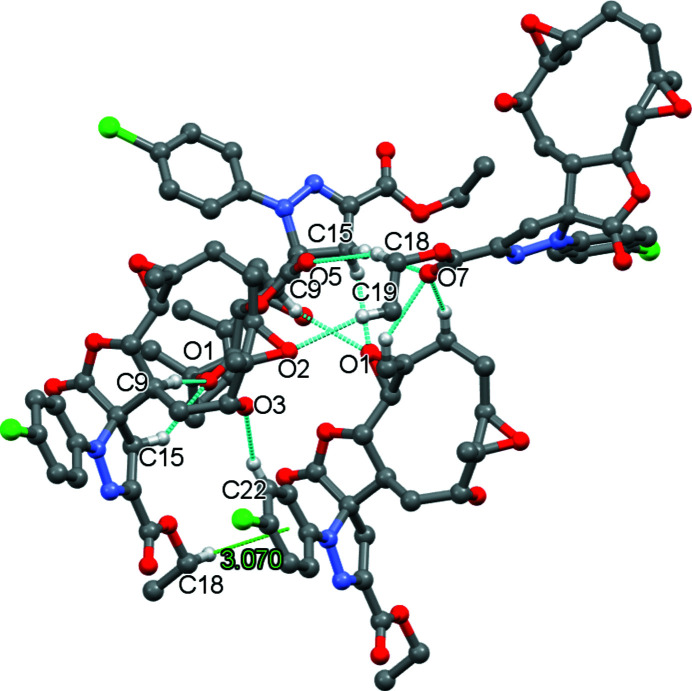
A projection showing the mol­ecules connected by hydrogen bonds (dashed cyan lines) and a C—H⋯π inter­action (dashed green line).

**Figure 3:**
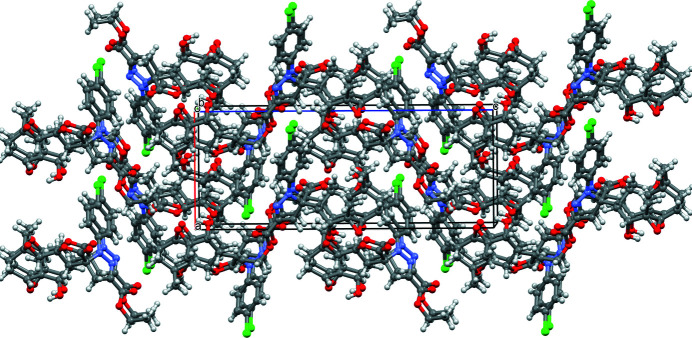
Crystal packing of the title compound showing the mol­ecules stacked approximately along the *b* axis.

In the crystal, the mol­ecules are linked together through five hydrogen bonds (Table 1 ▸) and one C—H⋯π inter­action to build an aggregate as shown in Fig. 2 ▸. An overall view of the crystal packing is shown in Fig. 3 ▸.

## Synthesis and crystallization

The title compound was obtained by the treatment of 9α-hy­droxy­parthenolide (500 mg) with *m*-chloro­perbenzoic acid (250 mg) in CH_2_Cl_2_ (75 ml). The mixture was stirred for 30 min at room temperature and treated with an aqueous solution of Na_2_CO_3_ (10%), then extracted with CH_2_Cl_2_. The residue obtained after evaporation of CH_2_Cl_2_ was chromatographed on a silica gel column with hexa­ne–ethyl acetate (60/40) as eluent to isolate 350 mg of 9α-hy­droxy-1β,10α-ep­oxy­parthenolide. To 300 mg of this compound dissolved in 50 ml of di­chloro­methane was added 1.2 equivalents of *N*-*para-*chloro­phenyl­hydrazono *α-*bromo­glyoxylate in the presence of 0.3 equivalents of caesium carbonate (Cs_2_CO_3_). The reaction mixture was stirred at room temperature for 3 h, and then the reaction was stopped by adding water (20 ml) and extracted three times with di­chloro­methane (3 × 30 ml). The organic phase was dried over sodium sulfate and then evaporated under vacuum. Chromatography of the residue obtained on silica gel column eluting with hexane ethyl acetate (70/30), allowed us to obtain the title compound in a 94% yield. Crystallization was carried out at room temperature from an ethyl acetate solution (m.p. 438–440 K).

## Refinement

Crystal data, data collection and structure refinement details are summarized in Table 2 ▸.

## Supplementary Material

Crystal structure: contains datablock(s) I. DOI: 10.1107/S2414314620009451/bt4095sup1.cif

Structure factors: contains datablock(s) I. DOI: 10.1107/S2414314620009451/bt4095Isup2.hkl

CCDC reference: 2015563

Additional supporting information:  crystallographic information; 3D view; checkCIF report

## Figures and Tables

**Table 1:** Hydrogen-bond geometry (Å, °) *Cg*5 is the centroid of the C20–C25 ring.

*D*—H⋯*A*	*D*—H	H⋯*A*	*D*⋯*A*	*D*—H⋯*A*
C3—H3*A*⋯O7^i^	0.97	2.51	3.340 (2)	144
C8—H8*B*⋯N2	0.97	2.52	2.928 (2)	105
C5—H5⋯O3	0.98	2.50	2.853 (2)	101
C9—H9⋯O3	0.98	2.35	2.850 (2)	111
C11—H11*A*⋯O7^i^	0.96	2.48	3.316 (3)	145
C15—H15*B*⋯O1^ii^	0.97	2.49	3.326 (2)	144
C19—H19*B*⋯O2^iii^	0.96	2.52	3.434 (3)	158
C21—H21⋯O4	0.93	2.53	3.340 (2)	145
C25—H25⋯N1	0.93	2.38	2.713 (2)	101
C22—H22⋯O3^iv^	0.93	2.49	3.295 (2)	146
O3—H3⋯O2	0.82	2.26	2.713 (2)	115
C18—H18*B*⋯*Cg*5^v^	0.97	3.07	3.436	104

Symmetry codes: (i) 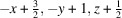 ; (ii) 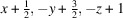 ; (iii) 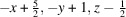 ; (iv) 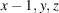 ; (v) 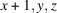 .

**Table 2:** Experimental details

Crystal data	
Chemical formula	C_25_H_29_ClN_2_O_7_
*M*_r_	504.95
Crystal system, space group	Orthorhombic, *P*2_1_2_1_2_1_
Temperature (K)	296
*a*, *b*, *c* (Å)	9.2324 (3), 11.1656 (4), 23.3497 (8)
*V* (Å^3^)	2407.01 (14)
*Z*	4
Radiation type	Mo *K*α
μ (mm^−1^)	0.21
Crystal size (mm)	0.37 × 0.29 × 0.22

Data collection	
Diffractometer	Bruker X8 APEX3
Absorption correction	Multi-scan (*SADABS*; Krause *et al.*, 2015 ▸)
*T*_min_, *T*_max_	0.680, 0.748
No. of measured, independent and observed [*I* > 2σ(*I*)] reflections	72348, 9629, 8323
*R*_int_	0.041
(sin θ/λ)_max_ (Å^−1^)	0.781

Refinement	
*R*[*F* ^2^ > 2σ(*F* ^2^)], *wR*(*F* ^2^), *S*	0.038, 0.107, 1.04
No. of reflections	9629
No. of parameters	319
H-atom treatment	H-atom parameters constrained
Δρ_max_, Δρ_min_ (e Å^−3^)	0.29, −0.20
Absolute structure	Flack *x* determined using 3371 quotients [(*I* ^+^)−(*I* ^−^)]/[(*I* ^+^)+(*I* ^−^)] (Parsons *et al.*, 2013 ▸)
Absolute structure parameter	0.009 (13)

Computer programs: *APEX3* and *SAINT* (Bruker, 2016 ▸), *SHELXT2014/5* (Sheldrick, 2015*a* ▸), *SHELXL2018/3* (Sheldrick, 2015*b* ▸), *WinGX* and *ORTEP-3 for Windows* (Farrugia, 2012 ▸), *Mercury* (Macrae *et al.*, 2020 ▸) and *publCIF* (Westrip, 2010 ▸).

## full crystallographic data

### Crystal data

**Table:**

C_25_H_29_ClN_2_O_7_	*D*_x_ = 1.393 Mg m^−^^3^
*M**_r_* = 504.95	Mo *K*α radiation, λ = 0.71073 Å
Orthorhombic, *P*2_1_2_1_2_1_	Cell parameters from 9629 reflections
*a* = 9.2324 (3) Å	θ = 2.5–33.7°
*b* = 11.1656 (4) Å	µ = 0.21 mm^−^^1^
*c* = 23.3497 (8) Å	*T* = 296 K
*V* = 2407.01 (14) Å^3^	Block, colourless
*Z* = 4	0.37 × 0.29 × 0.22 mm
*F*(000) = 1064	

### Data collection

**Table:**

Bruker X8 APEX3 diffractometer	9629 independent reflections
Radiation source: fine-focus sealed tube	8323 reflections with *I* > 2σ(*I*)
Graphite monochromator	*R*_int_ = 0.041
φ and ω scans	θ_max_ = 33.7°, θ_min_ = 2.5°
Absorption correction: multi-scan (SADABS; Krause *et al.*, 2015)	*h* = −14→14
*T*_min_ = 0.680, *T*_max_ = 0.748	*k* = −16→17
72348 measured reflections	*l* = −36→36

### Refinement

**Table:**

Refinement on *F*^2^	Hydrogen site location: inferred from neighbouring sites
Least-squares matrix: full	H-atom parameters constrained
*R*[*F*^2^ > 2σ(*F*^2^)] = 0.038	*w* = 1/[σ^2^(*F*_o_^2^) + (0.0657*P*)^2^ + 0.1273*P*] where *P* = (*F*_o_^2^ + 2*F*_c_^2^)/3
*wR*(*F*^2^) = 0.107	(Δ/σ)_max_ < 0.001
*S* = 1.04	Δρ_max_ = 0.29 e Å^−^^3^
9629 reflections	Δρ_min_ = −0.20 e Å^−^^3^
319 parameters	Absolute structure: Flack *x* determined using 3371 quotients [(*I*^+^)-(*I*^-^)]/[(*I*^+^)+(*I*^-^)] (Parsons *et al.*, 2013)
0 restraints	Absolute structure parameter: 0.009 (13)
Primary atom site location: structure-invariant direct methods	

### Special details

**Table:**

Geometry. All esds (except the esd in the dihedral angle between two l.s. planes) are estimated using the full covariance matrix. The cell esds are taken into account individually in the estimation of esds in distances, angles and torsion angles; correlations between esds in cell parameters are only used when they are defined by crystal symmetry. An approximate (isotropic) treatment of cell esds is used for estimating esds involving l.s. planes.

### Fractional atomic coordinates and isotropic or equivalent isotropic displacement parameters (Å^2^)

**Table:**

	*x*	*y*	*z*	*U*_iso_*/*U*_eq_	
C1	0.7150 (2)	0.67174 (15)	0.52147 (6)	0.0358 (3)	
H1	0.816146	0.696658	0.525874	0.043*	
C2	0.6528 (2)	0.61025 (18)	0.57192 (7)	0.0398 (4)	
C3	0.7498 (2)	0.59497 (19)	0.62349 (7)	0.0431 (4)	
H3A	0.691333	0.596845	0.657981	0.052*	
H3B	0.817534	0.661313	0.625230	0.052*	
C4	0.8344 (2)	0.4771 (2)	0.62139 (7)	0.0452 (4)	
H4A	0.903439	0.474656	0.652698	0.054*	
H4B	0.768135	0.410361	0.625931	0.054*	
C5	0.91385 (19)	0.46530 (17)	0.56510 (7)	0.0387 (3)	
H5	0.956502	0.540404	0.551415	0.046*	
C6	0.8807 (2)	0.37932 (14)	0.51919 (7)	0.0353 (3)	
C7	0.92537 (18)	0.40590 (14)	0.45781 (7)	0.0325 (3)	
H7	0.951281	0.328982	0.440387	0.039*	
C8	0.80988 (19)	0.46221 (13)	0.41933 (6)	0.0327 (3)	
H8A	0.717532	0.425418	0.428433	0.039*	
H8B	0.832484	0.441735	0.379960	0.039*	
C9	0.79126 (15)	0.59873 (13)	0.42294 (5)	0.0260 (2)	
H9	0.882322	0.631358	0.437827	0.031*	
C10	0.66934 (18)	0.64761 (13)	0.46090 (6)	0.0303 (3)	
H10	0.586034	0.593226	0.460247	0.036*	
C11	0.5270 (3)	0.5249 (3)	0.56737 (10)	0.0630 (7)	
H11A	0.471915	0.526959	0.602215	0.095*	
H11B	0.562651	0.445152	0.561103	0.095*	
H11C	0.466341	0.548048	0.535875	0.095*	
C12	0.7689 (4)	0.2822 (2)	0.52444 (10)	0.0658 (7)	
H12A	0.807943	0.208399	0.510044	0.099*	
H12B	0.684612	0.303666	0.502676	0.099*	
H12C	0.742773	0.272411	0.563958	0.099*	
C13	0.76350 (15)	0.65963 (12)	0.36420 (5)	0.0251 (2)	
C14	0.67465 (18)	0.77038 (14)	0.38029 (7)	0.0319 (3)	
C15	0.90386 (16)	0.69343 (14)	0.33170 (6)	0.0298 (3)	
H15A	0.899553	0.774632	0.317038	0.036*	
H15B	0.988681	0.684824	0.355864	0.036*	
C16	0.90345 (15)	0.60317 (14)	0.28410 (6)	0.0278 (2)	
C17	1.02307 (16)	0.57542 (14)	0.24392 (6)	0.0299 (3)	
C18	1.26216 (17)	0.62649 (17)	0.21770 (8)	0.0381 (3)	
H18A	1.275157	0.541298	0.211393	0.046*	
H18B	1.348372	0.656888	0.236473	0.046*	
C19	1.2428 (2)	0.6883 (2)	0.16137 (9)	0.0523 (5)	
H19A	1.166045	0.650405	0.140387	0.078*	
H19B	1.331107	0.683319	0.139785	0.078*	
H19C	1.218879	0.770902	0.167793	0.078*	
C20	0.55559 (14)	0.52443 (13)	0.32367 (6)	0.0268 (2)	
C21	0.44150 (17)	0.56677 (15)	0.35754 (7)	0.0333 (3)	
H21	0.453488	0.636501	0.378797	0.040*	
C22	0.31002 (16)	0.50546 (17)	0.35975 (7)	0.0355 (3)	
H22	0.235107	0.533222	0.382829	0.043*	
C23	0.29220 (15)	0.40303 (15)	0.32729 (7)	0.0327 (3)	
C24	0.40232 (18)	0.36072 (16)	0.29257 (8)	0.0376 (3)	
H24	0.388580	0.292186	0.270598	0.045*	
C25	0.53355 (17)	0.42143 (15)	0.29079 (7)	0.0341 (3)	
H25	0.607668	0.393186	0.267444	0.041*	
Cl1	0.12664 (5)	0.32803 (5)	0.32768 (2)	0.04545 (11)	
N1	0.78250 (14)	0.54795 (13)	0.27844 (5)	0.0302 (2)	
N2	0.68834 (13)	0.58649 (12)	0.31991 (5)	0.0294 (2)	
O1	0.6144 (2)	0.73304 (15)	0.55821 (6)	0.0593 (4)	
O2	1.0048 (2)	0.36152 (17)	0.55599 (7)	0.0592 (4)	
O3	1.05034 (14)	0.47806 (14)	0.45565 (7)	0.0471 (3)	
H3	1.077114	0.493426	0.488323	0.071*	
O4	0.63046 (15)	0.76298 (11)	0.43545 (5)	0.0379 (3)	
O5	0.64470 (18)	0.85384 (13)	0.35076 (6)	0.0503 (3)	
O6	1.13655 (12)	0.64598 (11)	0.25448 (5)	0.0344 (2)	
O7	1.01925 (16)	0.49953 (14)	0.20772 (6)	0.0481 (3)	

### Atomic displacement parameters (Å^2^)

**Table:**

	*U*^11^	*U*^22^	*U*^33^	*U*^12^	*U*^13^	*U*^23^
C1	0.0502 (9)	0.0350 (7)	0.0223 (6)	0.0008 (6)	0.0083 (6)	−0.0050 (5)
C2	0.0431 (8)	0.0529 (9)	0.0235 (6)	0.0019 (7)	0.0103 (6)	−0.0016 (6)
C3	0.0520 (10)	0.0554 (10)	0.0218 (6)	−0.0080 (8)	0.0058 (6)	−0.0037 (6)
C4	0.0549 (10)	0.0544 (10)	0.0262 (6)	−0.0055 (9)	−0.0030 (7)	0.0020 (7)
C5	0.0371 (7)	0.0463 (9)	0.0326 (7)	−0.0033 (7)	−0.0037 (6)	−0.0004 (6)
C6	0.0423 (8)	0.0345 (7)	0.0290 (6)	−0.0011 (6)	−0.0021 (6)	0.0044 (5)
C7	0.0360 (7)	0.0302 (6)	0.0312 (6)	0.0053 (5)	0.0047 (5)	0.0003 (5)
C8	0.0461 (8)	0.0270 (6)	0.0251 (6)	0.0038 (6)	−0.0025 (6)	−0.0038 (5)
C9	0.0321 (6)	0.0270 (6)	0.0189 (5)	0.0023 (5)	0.0027 (4)	−0.0017 (4)
C10	0.0386 (7)	0.0307 (6)	0.0214 (5)	0.0032 (5)	0.0066 (5)	−0.0009 (5)
C11	0.0445 (10)	0.108 (2)	0.0362 (9)	−0.0203 (12)	0.0057 (8)	0.0106 (11)
C12	0.102 (2)	0.0489 (11)	0.0470 (11)	−0.0351 (13)	0.0123 (12)	−0.0013 (9)
C13	0.0279 (5)	0.0279 (6)	0.0196 (5)	0.0006 (5)	0.0026 (4)	−0.0014 (4)
C14	0.0359 (7)	0.0314 (7)	0.0284 (6)	0.0045 (6)	0.0046 (5)	0.0004 (5)
C15	0.0310 (6)	0.0348 (7)	0.0237 (5)	−0.0048 (5)	0.0048 (5)	−0.0035 (5)
C16	0.0279 (6)	0.0362 (6)	0.0192 (5)	−0.0014 (5)	0.0029 (4)	−0.0010 (5)
C17	0.0291 (6)	0.0381 (7)	0.0225 (6)	−0.0017 (5)	0.0040 (5)	−0.0005 (5)
C18	0.0272 (6)	0.0469 (8)	0.0403 (8)	0.0042 (6)	0.0079 (6)	0.0079 (7)
C19	0.0459 (10)	0.0685 (13)	0.0424 (9)	−0.0081 (10)	0.0093 (8)	0.0156 (9)
C20	0.0235 (5)	0.0342 (6)	0.0227 (5)	0.0012 (5)	0.0004 (4)	0.0026 (5)
C21	0.0278 (6)	0.0410 (8)	0.0311 (7)	0.0008 (6)	0.0032 (5)	−0.0049 (6)
C22	0.0254 (6)	0.0477 (8)	0.0335 (7)	0.0014 (6)	0.0035 (5)	−0.0017 (6)
C23	0.0258 (5)	0.0414 (7)	0.0309 (6)	−0.0026 (5)	−0.0030 (5)	0.0067 (6)
C24	0.0344 (7)	0.0391 (8)	0.0393 (8)	−0.0029 (6)	−0.0002 (6)	−0.0058 (6)
C25	0.0287 (6)	0.0376 (7)	0.0360 (7)	0.0008 (6)	0.0035 (5)	−0.0052 (6)
Cl1	0.03149 (17)	0.0572 (3)	0.0476 (2)	−0.01126 (17)	0.00022 (16)	0.00024 (19)
N1	0.0288 (5)	0.0417 (6)	0.0200 (4)	−0.0019 (5)	0.0030 (4)	−0.0039 (4)
N2	0.0257 (5)	0.0410 (6)	0.0213 (5)	−0.0025 (5)	0.0030 (4)	−0.0052 (4)
O1	0.0854 (12)	0.0599 (9)	0.0325 (6)	0.0294 (9)	0.0141 (7)	−0.0095 (6)
O2	0.0627 (9)	0.0719 (10)	0.0430 (7)	0.0267 (8)	−0.0159 (7)	−0.0009 (7)
O3	0.0330 (6)	0.0561 (8)	0.0522 (8)	−0.0009 (5)	0.0109 (6)	0.0037 (6)
O4	0.0488 (6)	0.0357 (5)	0.0292 (5)	0.0145 (5)	0.0090 (5)	−0.0007 (4)
O5	0.0651 (9)	0.0426 (7)	0.0433 (7)	0.0187 (7)	0.0090 (6)	0.0122 (6)
O6	0.0281 (5)	0.0439 (6)	0.0311 (5)	−0.0027 (4)	0.0051 (4)	−0.0025 (4)
O7	0.0467 (7)	0.0599 (8)	0.0378 (6)	−0.0109 (6)	0.0138 (5)	−0.0204 (6)

### Geometric parameters (Å, º)

**Table:**

C1—O1	1.438 (2)	C12—H12C	0.9600
C1—C2	1.479 (2)	C13—N2	1.4893 (18)
C1—C10	1.500 (2)	C13—C14	1.531 (2)
C1—H1	0.9800	C13—C15	1.5484 (19)
C2—O1	1.452 (3)	C14—O5	1.192 (2)
C2—C11	1.506 (3)	C14—O4	1.3535 (19)
C2—C3	1.510 (3)	C15—C16	1.500 (2)
C3—C4	1.531 (3)	C15—H15A	0.9700
C3—H3A	0.9700	C15—H15B	0.9700
C3—H3B	0.9700	C16—N1	1.2823 (19)
C4—C5	1.511 (3)	C16—C17	1.4819 (19)
C4—H4A	0.9700	C17—O7	1.198 (2)
C4—H4B	0.9700	C17—O6	1.3339 (19)
C5—O2	1.447 (2)	C18—O6	1.4594 (19)
C5—C6	1.471 (2)	C18—C19	1.496 (3)
C5—H5	0.9800	C18—H18A	0.9700
C6—O2	1.446 (2)	C18—H18B	0.9700
C6—C12	1.502 (3)	C19—H19A	0.9600
C6—C7	1.521 (2)	C19—H19B	0.9600
C7—O3	1.408 (2)	C19—H19C	0.9600
C7—C8	1.530 (2)	C20—C25	1.398 (2)
C7—H7	0.9800	C20—C21	1.399 (2)
C8—C9	1.536 (2)	C20—N2	1.4107 (17)
C8—H8A	0.9700	C21—C22	1.395 (2)
C8—H8B	0.9700	C21—H21	0.9300
C9—C10	1.5333 (19)	C22—C23	1.382 (3)
C9—C13	1.5522 (18)	C22—H22	0.9300
C9—H9	0.9800	C23—C24	1.383 (2)
C10—O4	1.4635 (19)	C23—Cl1	1.7429 (15)
C10—H10	0.9800	C24—C25	1.389 (2)
C11—H11A	0.9600	C24—H24	0.9300
C11—H11B	0.9600	C25—H25	0.9300
C11—H11C	0.9600	N1—N2	1.3706 (16)
C12—H12A	0.9600	O3—H3	0.8200
C12—H12B	0.9600		
			
O1—C1—C2	59.68 (12)	C6—C12—H12A	109.5
O1—C1—C10	117.82 (16)	C6—C12—H12B	109.5
C2—C1—C10	123.95 (16)	H12A—C12—H12B	109.5
O1—C1—H1	114.7	C6—C12—H12C	109.5
C2—C1—H1	114.7	H12A—C12—H12C	109.5
C10—C1—H1	114.7	H12B—C12—H12C	109.5
O1—C2—C1	58.73 (11)	N2—C13—C14	111.33 (12)
O1—C2—C11	113.2 (2)	N2—C13—C15	100.56 (10)
C1—C2—C11	122.45 (16)	C14—C13—C15	111.83 (12)
O1—C2—C3	115.30 (16)	N2—C13—C9	116.76 (12)
C1—C2—C3	117.21 (16)	C14—C13—C9	103.04 (10)
C11—C2—C3	116.23 (17)	C15—C13—C9	113.68 (11)
C2—C3—C4	111.96 (15)	O5—C14—O4	121.89 (15)
C2—C3—H3A	109.2	O5—C14—C13	127.88 (14)
C4—C3—H3A	109.2	O4—C14—C13	110.23 (12)
C2—C3—H3B	109.2	C16—C15—C13	101.37 (11)
C4—C3—H3B	109.2	C16—C15—H15A	111.5
H3A—C3—H3B	107.9	C13—C15—H15A	111.5
C5—C4—C3	110.50 (15)	C16—C15—H15B	111.5
C5—C4—H4A	109.6	C13—C15—H15B	111.5
C3—C4—H4A	109.6	H15A—C15—H15B	109.3
C5—C4—H4B	109.6	N1—C16—C17	118.89 (13)
C3—C4—H4B	109.6	N1—C16—C15	113.68 (12)
H4A—C4—H4B	108.1	C17—C16—C15	127.41 (12)
O2—C5—C6	59.40 (12)	O7—C17—O6	124.86 (14)
O2—C5—C4	118.64 (16)	O7—C17—C16	124.96 (14)
C6—C5—C4	126.13 (16)	O6—C17—C16	110.18 (12)
O2—C5—H5	113.8	O6—C18—C19	110.71 (15)
C6—C5—H5	113.8	O6—C18—H18A	109.5
C4—C5—H5	113.8	C19—C18—H18A	109.5
O2—C6—C5	59.45 (12)	O6—C18—H18B	109.5
O2—C6—C12	113.36 (17)	C19—C18—H18B	109.5
C5—C6—C12	123.69 (16)	H18A—C18—H18B	108.1
O2—C6—C7	111.83 (15)	C18—C19—H19A	109.5
C5—C6—C7	120.19 (14)	C18—C19—H19B	109.5
C12—C6—C7	113.85 (16)	H19A—C19—H19B	109.5
O3—C7—C6	111.58 (14)	C18—C19—H19C	109.5
O3—C7—C8	108.36 (13)	H19A—C19—H19C	109.5
C6—C7—C8	116.41 (14)	H19B—C19—H19C	109.5
O3—C7—H7	106.7	C25—C20—C21	118.60 (13)
C6—C7—H7	106.7	C25—C20—N2	119.77 (13)
C8—C7—H7	106.6	C21—C20—N2	121.55 (13)
C7—C8—C9	116.98 (13)	C22—C21—C20	120.68 (15)
C7—C8—H8A	108.1	C22—C21—H21	119.7
C9—C8—H8A	108.1	C20—C21—H21	119.7
C7—C8—H8B	108.1	C23—C22—C21	119.32 (14)
C9—C8—H8B	108.1	C23—C22—H22	120.3
H8A—C8—H8B	107.3	C21—C22—H22	120.3
C10—C9—C8	117.84 (13)	C22—C23—C24	121.10 (14)
C10—C9—C13	103.52 (11)	C22—C23—Cl1	119.95 (12)
C8—C9—C13	113.88 (11)	C24—C23—Cl1	118.92 (13)
C10—C9—H9	107.0	C23—C24—C25	119.47 (15)
C8—C9—H9	107.0	C23—C24—H24	120.3
C13—C9—H9	107.0	C25—C24—H24	120.3
O4—C10—C1	107.08 (12)	C24—C25—C20	120.81 (14)
O4—C10—C9	104.98 (11)	C24—C25—H25	119.6
C1—C10—C9	113.75 (13)	C20—C25—H25	119.6
O4—C10—H10	110.3	C16—N1—N2	109.17 (12)
C1—C10—H10	110.3	N1—N2—C20	116.16 (12)
C9—C10—H10	110.3	N1—N2—C13	111.55 (11)
C2—C11—H11A	109.5	C20—N2—C13	129.12 (11)
C2—C11—H11B	109.5	C1—O1—C2	61.59 (11)
H11A—C11—H11B	109.5	C6—O2—C5	61.15 (11)
C2—C11—H11C	109.5	C7—O3—H3	109.5
H11A—C11—H11C	109.5	C14—O4—C10	111.49 (11)
H11B—C11—H11C	109.5	C17—O6—C18	115.30 (13)

### Hydrogen-bond geometry (Å, º)

*Cg*5 is the centroid of the C20–C25 ring.

**Table:**

*D*—H···*A*	*D*—H	H···*A*	*D*···*A*	*D*—H···*A*
C3—H3*A*···O7^i^	0.97	2.51	3.340 (2)	144
C8—H8*B*···N2	0.97	2.52	2.928 (2)	105
C5—H5···O3	0.98	2.50	2.853 (2)	101
C9—H9···O3	0.98	2.35	2.850 (2)	111
C11—H11*A*···O7^i^	0.96	2.48	3.316 (3)	145
C15—H15*B*···O1^ii^	0.97	2.49	3.326 (2)	144
C19—H19*B*···O2^iii^	0.96	2.52	3.434 (3)	158
C21—H21···O4	0.93	2.53	3.340 (2)	145
C25—H25···N1	0.93	2.38	2.713 (2)	101
C22—H22···O3^iv^	0.93	2.49	3.295 (2)	146
O3—H3···O2	0.82	2.26	2.713 (2)	115
C18—H18*B*···*Cg*5^v^	0.97	3.07	3.436	104

Symmetry codes: (i) −*x*+3/2, −*y*+1, *z*+1/2; (ii) *x*+1/2, −*y*+3/2, −*z*+1; (iii) −*x*+5/2, −*y*+1, *z*−1/2; (iv) *x*−1, *y*, *z*; (v) *x*+1, *y*, *z*.
